# Female Sex Is Not a Uniform Risk Factor in Atrial Fibrillation

**DOI:** 10.1016/j.jacadv.2026.102826

**Published:** 2026-06-03

**Authors:** Cecile McGarvey, Emma Lunn, Yishi Jia, Yara Menassa, Yingshuo Liu, Mayana Bsoul, Christian Massad, Maximillian Moersdorf, Alex El Darazi, Michel Abou Khalil, Mohammad M. Atasi, Carlo El Khoury, Rodolfo Montiel Quintero, Omar Kriedieh, Qussay Marashly, Nassir F. Marrouche, Amitabh C. Pandey, Han Feng

**Affiliations:** Tulane Research Innovation for Arrhythmia Discovery, Tulane University School of Medicine, Department of Cardiology, New Orleans, Louisiana, USA

**Keywords:** arterial embolism, atrial fibrillation, female, thromboembolic stroke

## Abstract

**Background:**

Female sex is a part of the stroke risk stratification in atrial fibrillation (AF), although emerging evidence suggests it may function as a risk modifier rather than an independent risk factor.

**Objectives:**

This study aimed to determine whether female sex is a risk factor or risk modifier for stroke in patients with AF.

**Methods:**

Using TriNetX, nonvalvular AF patients were identified and stratified by sex and age (<65, 65-74, and ≥75 years). Female and male patients were propensity matched for comorbidities and anticoagulation. Analyses were conducted comparing male and female patients with no CHA_2_DS_2_-VA risk factors beyond age and with one additional CHA_2_DS_2_-VA risk factor. The risks of stroke and arterial embolism were compared over a 1-year follow-up period.

**Results:**

In patients without additional CHA_2_DS_2_-VA risk factors or anticoagulation (n = 252,528), female sex was associated with increased stroke risk only among patients aged ≥75 years (HR: 1.244; 95% CI: 1.087-1.423; *P* = 0.001). Similarly, in patients with one additional CHA_2_DS_2_-VA risk factor (n = 607,612), stroke risk was increased among female patients ≥75 years (HR: 1.065; 95% CI: 1.014-1.118; *P* = 0.012).

**Conclusions:**

Female sex acts as a modest risk modifier for thromboembolic stroke. Increased risk is primarily observed in patients with greater comorbidity burden or advanced age (≥75 years).

Atrial fibrillation (AF) is the most common cardiac arrhythmia, with one of the most feared complications being thromboembolic stroke.^1^ Despite the increased incidence and prevalence of AF in men, the absolute number of patients ≥75 years old with AF is greater in female populations due to overall longer survival.^2^ For years, several studies have demonstrated an increased risk of thromboembolic stroke among female AF patients when compared to male patients.^3^^,^^4^ Studies have also investigated the increased severity, poorer post-stroke outcomes among female populations,^5^^,^^6^ and overall poorer quality of life before and after intervention.^7^ For these reasons, female sex has been included as a category in the risk stratification CHA_2_DS_2_-VASc score, contributing to clinical decision-making about anticoagulation status since 2010.^8^

Despite the historic consideration of female sex as a significant stroke risk factor in AF patients, female patients are largely underrepresented in AF studies, comprising only about one-third of AF randomized controlled trial study populations.^9^ More recent studies have suggested that female sex is a risk modifier rather than a risk factor for thromboembolic stroke in AF patients. As a modifier, female sex increases the risk of stroke among older patients ≥75 but does not have the same effect in younger populations.^10^ From these new studies, the European Society of Cardiology has modified its guidelines to promote use of the CHA_2_DS_2_-VA score, excluding female sex as a contributory category.^11^ In accordance with these guidelines, younger (<75) female patients without any additional risk factors are not recommended to be on anticoagulation. Some studies have even marginally favored the predictive capabilities of this modified CHA_2_DS_2_-VA score over the traditional CHA_2_DS_2_-VASc score.^12^

The most updated version of the American Heart Association/American College of Cardiology/American College of Clinical Pharmacy/Heart Rhythm Society guidelines for AF diagnosis and management continue to recommend the use of the CHA_2_DS_2_-VASc score when determining anticoagulation status for patients. Men with a score of 1 and women with a score of 2 have a class 2a recommendation to start anticoagulation^13^—this means that a female AF patient over 65 ultimately qualifies for anticoagulation. However, this may result in overtreatment of female patients with anticoagulation, which is not without its risks.

In this retrospective cohort study, we investigate our hypothesis that previous discrepancies between female sex as a risk factor vs a risk modifier for stroke in AF patients are due to the heterogeneity of female populations. With a large electronic health record (EHR) database available, we will quantitatively test our hypothesis to determine the exact role of female sex in AF stroke risk.

## Methods

### Study data source

This observational cohort study was conducted using the TriNetX database, a global multi-health care organization database. TriNetX collects deidentified patient data from multiple health care organizations within the United States and around the world, extracting information from EHRs. Deidentified patient data, including demographics, ICD-9 and ICD-10 diagnosis codes, medications, laboratory values, and procedures, are queried in the database. The dates of data collection ranged from 2015 to 2025.

TriNetX is compliant with the Health Insurance Portability and Accountability Act Privacy Rule and U.S. Federal Regulations, ensuring patient confidentiality. With patient data completely deidentified, ethics approval is not required prior to use of the database. For more information about the TriNetX database, see the following website https://trinetx.com/data-sets-analytics/.

### Study population

Patients with nonvalvular AF were identified using the TriNetX database. Patients with paroxysmal, persistent, chronic, or unspecified AF were included in this study (ICD-10-CM codes I48.0, I48.1, I48.2, or I48.91). Nonvalvular AF was defined as the exclusion of patients with rheumatic or nonrheumatic mitral valve disease (ICD-10-CM codes I05.0, I05.8, I34.0, I34.1, or I34.2). See “Supplemental Information” for ICD-10-CM codes used in exclusion criteria.

### Study design

**Figure 1:**
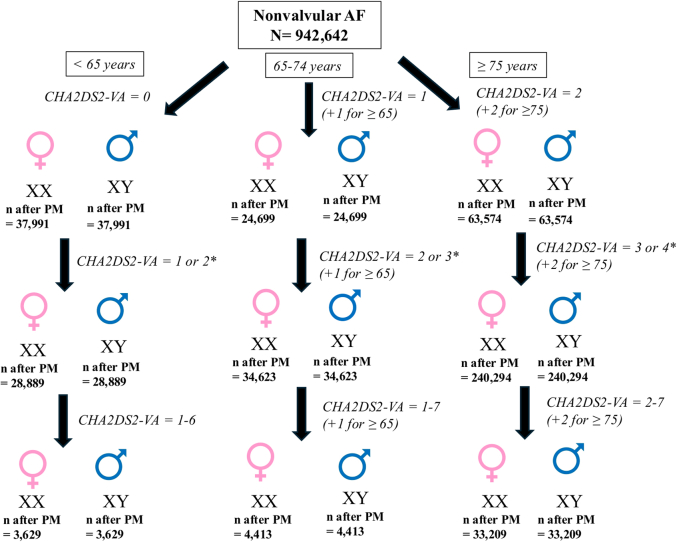
**Study Design and Population** Diagram showing the way subgroups were organized. Our study included nonvalvular atrial fibrillation patients who were further subdivided by age group (<65, 65-74, and ≥75) and sex (female vs male). Female and male patients in each category were then propensity matched in a 1:1 ratio for age, comorbidities, and anticoagulation status to be used in analysis. AF = atrial fibrillation; PM = propensity matching.

As shown in Figure 1, nonvalvular AF patients were divided into 6 groups based on sex (male vs female) and age (<65 years, 65-74 years, ≥75 years). In our study, we categorized female and male patients according to their biological sex assigned at birth as designated in the EHR. Male and female patients without additional CHA_2_DS_2_-VA risk factors beyond age (<65 years: CHA_2_DS_2_-VA = 0; 65-74 years: CHA_2_DS_2_-VA = 1; ≥75 years: CHA_2_DS_2_-VA = 2) and not receiving anticoagulation therapy were compared after propensity matching, as described in the Statistical Analyses section. Patients treated with warfarin, apixaban, edoxaban, rivaroxaban, or dabigatran were excluded from this analysis. Likewise, patients with essential hypertension (HTN), congestive heart failure, type 2 diabetes mellitus (DM), prior myocardial infarction (MI), prior transient ischemic attack (TIA), or other peripheral vascular diseases were excluded from this analysis. In this way, male and female patients without anticoagulation or additional thromboembolic risk factors were compared in each age category.

A similar subanalysis was conducted in male and female patients with either DM, HTN, peripheral vascular disease, prior MI, prior TIA, or heart failure. Prior TIA counts for 2 points, whereas all other risk factors count for 1 point in the CHA_2_DS_2_-VA risk stratification tool. In this subanalysis, patients had one additional CHA_2_DS_2_-VA risk factor in addition to age (<65 years: CHA_2_DS_2_-VA = 1 or 2; 65-74 years: CHA_2_DS_2_-VA = 2 or 3; ≥75 years: CHA_2_DS_2_-VA = 3 or 4).

A sensitivity analysis was performed to isolate nonvalvular AF patients who were on anticoagulation. Patients with a current prescription of apixaban, rivaroxaban, edoxaban, dabigatran, or warfarin were included in this subanalysis, while those without such prescription were excluded. These patients had CHA_2_DS_2_-VA scores ranging from 1 to 8, and each risk factor was propensity matched between groups. Like the above analyses, male and female patients were compared in each age category.

### Study outcomes

The primary outcome of this study was the occurrence of cerebral infarction (ICD-10-CM I63) or arterial embolism and thrombosis (ICD-10-CM I74) during a 1-year follow-up period, in accordance with the annual predictive risk of thromboembolic stroke as determined by the CHA_2_DS_2_-VASc score. Patients with the primary outcome occurring prior to the time window were excluded from analysis.

### Statistical analyses

Male and female patients with nonvalvular AF and the exclusion criteria of anticoagulation and CHA_2_DS_2_-VASc risk factors were propensity matched in a 1:1 ratio using nearest-neighbor matching based on the following characteristics: atherosclerotic heart disease, ischemic cardiomyopathy, tobacco use, obstructive sleep apnea, and body mass index, with balance assessed by standardized mean differences (<0.1). Then, male and female nonvalvular AF patients with one additional CHA_2_DS_2_-VASc factor besides age, either DM, HTN, peripheral vascular disease, prior MI, prior TIA, or heart failure, were propensity matched. Within this subanalysis, patients on anticoagulation were excluded in the <65 years (CHA_2_DS_2_-VA = 1 or 2) and 65 to 74 years (CHA_2_DS_2_-VA = 2 or 3) groups. In patients aged ≥75 years (CHA_2_DS_2_-VA = 3 or 4), anticoagulation was included in propensity matching. Subjects with missing data in key variables (including diagnosis, age, and sex) were excluded from the analysis. In the sensitivity analysis including only anticoagulated patients, CHA_2_DS_2_-VA risk factors including congestive heart failure, HTN, TIA, diabetes, and vascular disease history were propensity matched in a 1:1 ratio. The type of anticoagulation, including apixaban, rivaroxaban, edoxaban, dabigatran, or warfarin, was also 1:1 propensity matched between groups.

Age-stratified analyses were prespecified and conducted across 3 clinically relevant age groups (<65, 65-74, ≥75), reflecting their differential contribution to the CHA_2_DS_2_-VASc score. Propensity score matching was performed separately within each age stratum, using the same covariates as described above, rather than across pooled cohorts, to ensure appropriate balance within each stratum. This approach was selected to preserve comparability across groups while accommodating the TriNetX platform's matching capabilities.

All statistical analyses were performed using the TriNetX database statistical platform. Categorical variables were compared via chi-square tests, and continuous variables were compared via 2-sample *t*-tests. Kaplan-Meier curves and log-rank tests were conducted to compare the groups. Cox regression models were further developed to estimate the HRs and their 95% CIs between groups, adjusting for other clinically relevant covariates. A 2-sided 0.05 was considered significant.

## Results

### Patients aged <65 years

#### CHA_2_DS_2_-VA: 0

A total of 98,135 patients aged <65 without anticoagulation and with a CHA_2_DS_2_-VA score of 0 were identified, including 37,993 (38.7%) female patients. After matching for age and non-CHA_2_DS_2_-VA comorbidities, 37,991 female patients were matched to an equal number of male patients (Supplemental Table 1).

**Figure 2:**
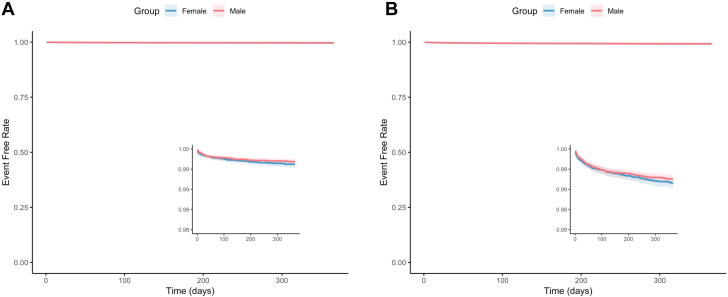
**Kaplan-Meier for Thromboembolic Stroke Risk, <65 Cohort** (A) Kaplan-Meier survival analysis curve showing no difference (*P* = 0.200) in stroke over time between female and male patients <65 years old without any additional stroke risk factors (CHA_2_DS_2_-VA = 0) and who were not currently on any anticoagulation. (B) Kaplan-Meier survival analysis curve comparing occurrence of stroke over time between female and male patients <65 years old with CHA_2_DS_2_-VA = 1 or 2. There was no significant difference in risk of stroke between female and male cohorts (*P* = 0.236).

During a 1-year follow-up, no significant sex-based differences were observed in thromboembolic stroke (HR: 1.201; 95% CI: 0.907-1.589; *P* = 0.200) or arterial embolism (HR: 0.642; 95% CI: 0.339-1.215; *P* = 0.170). Kaplan-Meier curves can be seen in Figure 2A.

#### CHA_2_DS_2_-VA: 1 or 2

82,687 patients <65 with one CHA_2_DS_2_-VA risk factor were identified (CHA_2_DS_2_-VA: 1 or 2), including 29,081 (35.2%) female patients. After propensity matching for age and non-CHA_2_DS_2_-VA risk factors, 28,889 male and 28,889 female patients were compared (Supplemental Material 1).

During a 1-year follow-up, there were no significant differences in thromboembolic stroke (HR: 1.136; 95% CI: 0.920-1.403; *P* = 0.236) or arterial embolism (HR: 1.079; 95% CI: 0.675-1.726; *P* = 0.749), shown in Figure 2B.

#### Anticoagulated and CHA_2_DS_2_-VA: 1 to 6

After propensity matching, we identified 3,269 female AF patients and an equal number of male patients, all of which had a current anticoagulation prescription. Over 1 year, there was no significant difference in stroke risk (HR: 1.090; 95% CI: 0.895-1.327; *P* = 0.393) or arterial embolism (HR: 1.234; 95% CI: 0.825-1.844; *P* = 0.306) (Supplemental Figure 1a).

### Patients aged 65 to 74 years

#### CHA_2_DS_2_-VA: 1

A total of 62,332 patients aged 65 to 74 years without anticoagulation or CHA_2_DS_2_-VA risk factors aside from age were identified, including 24,702 (39.6%) female patients. After matching, 24,699 female and 24,699 male patients were included (Supplemental Table 2).

**Figure 3:**
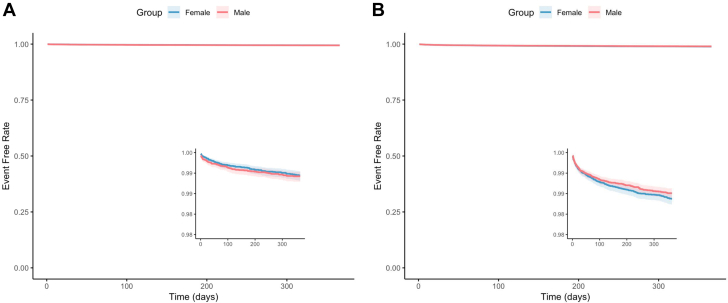
**Kaplan-Meier for Thromboembolic Stroke Risk, 65 to 74 Cohort** (A) Kaplan-Meier survival analysis curve showing no difference (*P* = 0.512) in occurrence of stroke over time between female and male patients aged 65 to 74 years old without any additional stroke risk factors (CHA_2_DS_2_-VA = 1, +1 point for age) and without any anticoagulation. (B) Kaplan-Meier survival analysis curve comparing occurrence of stroke over time between female and male patients aged 65 to 74 years old with CHA_2_DS_2_-VA score of 2 or 3 (+1 for age, +1 or +2 for additional risk factor). Patients were propensity matched for other risk factors, including obstructive sleep apnea, tobacco use, atherosclerotic heart disease, and ischemic cardiomyopathy. There was no sex-based difference in stroke risk in this age group (*P* = 0.167).

No significant sex-based differences were observed in thromboembolic stroke risk (HR: 0.909; 95% CI: 0.683-1.210; *P* = 0.512) or arterial embolism (HR: 0.835; 95% CI: 0.417-1.673; *P* = 0.611) during 1-year follow-up (Figure 3A).

#### CHA_2_DS_2_-VA: 2 or 3

94,060 patients aged 65 to 74 years old with one additional CHA_2_DS_2_-VA risk factor beyond age were identified, with 34,817 (37.0%) female patients. After propensity matching, 34,623 female patients were compared to an equal number of male patients.

After a 1-year follow-up period, there were no sex-based differences in thromboembolic stroke (HR: 1.128; 95% CI: 0.951-1.338; *P* = 0.167) or arterial embolism (HR: 1.037; 95% CI: 0.644-1.668; *P* = 0.967), shown in Figure 3B.

#### Anticoagulated and CHA_2_DS_2_-VA: 1 to 7

A total of 4,413 female nonvalvular AF patients aged 65 to 74 years old with a current anticoagulation prescription were propensity matched to an equal number of male patients. There was no significant difference in thromboembolic stroke risk (HR: 0.944; 95% CI: 0.792-1.125; *P* = 0.517) or arterial embolism risk (HR: 0.959; 95% CI: 0.619-1.487; *P* = 0.853) over a 1-year period between groups. (Supplemental Figure 1b).

### Patients aged ≥75 years

#### CHA_2_DS_2_-VA: 2

**Figure 4:**
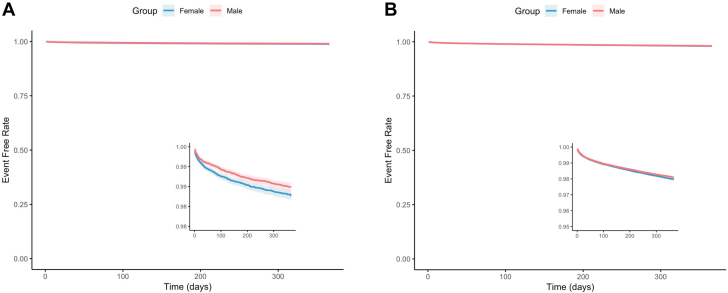
**Kaplan-Meier for Thromboembolic Stroke Risk, ≥75 Cohort** (A) Kaplan-Meier survival analysis curve showing significantly increased risk of stroke over time (*P* = 0.001) in female patients aged ≥75 years old without any additional stroke risk factors (CHA_2_DS_2_-VA = 2, including +2 points for age). (B) Kaplan-Meier survival analysis of male and female patients ≥75 years with one additional CHA_2_DS_2_-VA risk factor (CHA_2_DS_2_-VA = 3 or 4). Unlike younger age groups in the risk modifier analysis, females ≥75 had significantly greater risk of stroke compared to male patients (*P* = 0.012).

In contrast to younger cohorts, among 127,148 patients aged ≥75 years without anticoagulation or additional CHA_2_DS_2_-VA risk factors (63,574 female and 63,574 male patients after matching) (Supplemental Table 3), female sex was independently associated with a higher risk of thromboembolic stroke (HR: 1.244; 95% CI: 1.087-1.423; *P* = 0.001). Stroke occurred in 476 female patients (0.783%) and 382 male patients (0.624%), resulting in an absolute risk reduction of 0.159%, or about 1 extra stroke per 629 patients. No difference was observed in arterial embolism (HR: 0.963; 95% CI: 0.669-1.386; *P* = 0.840). Kaplan-Meier curves are shown in Figure 4A.

#### CHA_2_DS_2_-VA: 3 or 4

A total of 568,109 patients aged ≥75 with one additional risk factor (CHA_2_DS_2_-VA: 3 or 4) were identified, with 281,078 (49.5%) of the patients being female. After propensity matching for comorbidities and anticoagulation status, 240,294 female patients were compared to an equal number of male patients. Female sex was independently associated with an increased risk of thromboembolic stroke (HR: 1.065; 95% CI: 1.014-1.118; *P* = 0.012), as shown in Figure 4B. Stroke was observed in 3,337 (1.526%) female patients compared to 3,152 male patients (1.421%), resulting in an absolute risk reduction of 0.105%, or about 1 extra stroke per 952 patients. There was no difference in arterial embolism (HR: 1.003; 95% CI: 0.866-1.162; *P* = 0.967).

#### Anticoagulated and CHA_2_DS_2_-VA: 2 to 8

In the ≥75 years old cohort, 33,209 female patients were compared to an equal number of male patients. There was a significant difference between cohorts, with female patients having an increased 1-year risk of stroke (HR: 1.071; 95% CI: 1.004-1.143; *P* = 0.037). Stroke was observed in 1868 female patients and 1791 male patients, resulting in an absolute risk reduction of 0.005%, or about 1 extra stroke per 2000 patients. There was no difference in arterial embolism (HR: 0.973; 95% CI: 0.801-1.183; *P* = 0.785). (Supplemental Figure 1c).

## Discussion

In patients without additional CHA_2_DS_2_-VA risk factors and without anticoagulation, there was no sex difference in stroke or embolism risk between younger patients (<65 and 65-74 cohorts). However, older female patients (≥75 years) demonstrated a significant, yet modest, increased risk of stroke compared to male counterparts (as shown in the Central Illustration). By excluding patients with CHA_2_DS_2_-VA risk factors, we aimed to determine if female sex is an independent risk factor. Given its lack of association with increased stroke risk in younger cohorts (<65 and 65-74), it seems female sex is a risk modifier, modestly increasing stroke risk only when in the presence of older age (≥75), as summarized in Table 1.

**Central Illustration:**
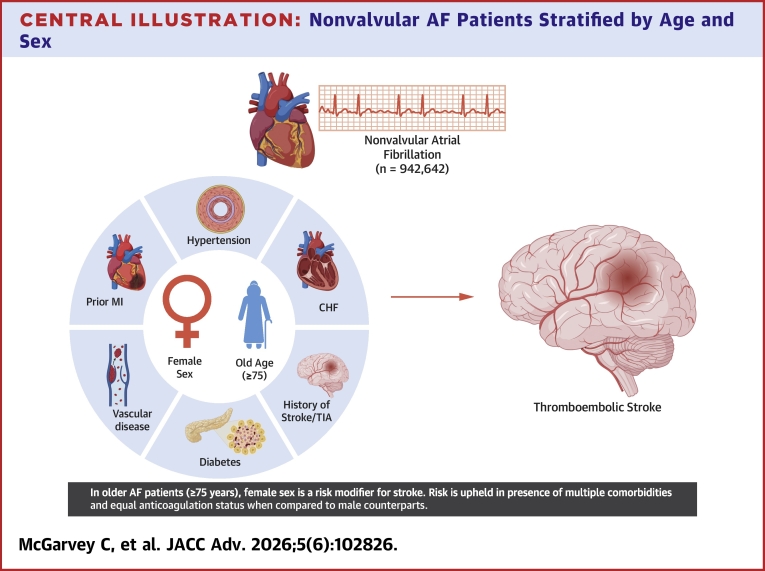
**Nonvalvular AF Patients Stratified by Age and Sex** After propensity matching for comorbidities and anticoagulation, 1-year stroke risk was compared in female and male patients. Female sex modified stroke risk in presence of greater comorbidity burden and older age (≥75 years old). CHF = congestive heart failure; MI = myocardial infarction; TIA = transient ischemic attack; other abbreviation as in Figure 1.

**Table 1:** Risk of Cerebral Infarction and Arterial Embolism by Age and CHA_2_DS_2_-VA Score

	Thromboembolic Stroke: HR	95% CI	*P* Value	Arterial Embolism: HR	95% CI	*P* Value
Age <65 years (CHA_2_DS_2_-VA: 0)	1.201	0.907-1.589	0.200	0.642	0.339-1.215	0.170
Age 65-74 years (CHA_2_DS_2_-VA: 1)	0.909	0.683-1.210	0.512	0.835	0.417-1.673	0.611
Age ≥75 years (CHA_2_DS_2_-VA: 2)	1.244	1.087-1.423	0.001∗∗	0.963	0.669-1.386	0.840
Age <65 years (CHA_2_DS_2_-VA: 1 or 2)	1.136	0.920-1.403	0.236	1.079	0.675-1.726	0.749
Age 65-74 years (CHA_2_DS_2_-VA: 2)	1.128	0.951-1.338	0.167	1.037	0.644-1.668	0.967
Age ≥75 years (CHA_2_DS_2_-VA: 3)	1.065	1.014-1.118	0.012∗∗	1.003	0.866-1.162	0.967

Risk of thromboembolic stroke and arterial embolism in female patients without anticoagulation and stratified by CHA_2_DS_2_-VA score compared to male patients after propensity matching in 1:1 ratio. Female patients in the ≥75 cohort only were at significantly increased risk of thromboembolic stroke compared to male counterparts. There was no significant difference in thromboembolic stroke between groups in younger cohorts (age <75 years), and there was no significant difference in arterial embolism between groups in any cohort.

Sensitivity analysis investigating stroke risk among anticoagulated patients further supported our conclusion of female sex as a risk modifier. Among anticoagulated patients <75 years old, there was no significant difference in stroke risk between male and female patients after matching for comorbidities, as summarized in Table 2. However, among older patients, female sex seemed to modify stroke risk, with female patients in this age category having a modestly increased risk of stroke despite equal anticoagulation when compared to male patients.

**Table 2:** Risk of Thromboembolic Stroke and Arterial Embolism in Anticoagulated Sensitivity Analysis

	Thromboembolic Stroke: HR	95% CI	*P* Value	Arterial Embolism: HR	95% CI	*P* Value
Age <65 years (CHA_2_DS_2_-VA: 1-6)	1.090	0.895-1.327	0.393	1.234	0.825-1.844	0.360
Age 65-74 years (CHA_2_DS_2_-VA: 1-7)	0.944	0.792-1.125	0.517	0.959	0.619-1.487	0.853
Age ≥75 years (CHA_2_DS_2_-VA: 2-8)	1.071	1.004-1.143	0.037∗∗	0.973	0.801-1.183	0.785

Risk of thromboembolic stroke and arterial embolism in cohort of patients on current anticoagulation stratified by age. Female patients in ≥75 years cohort were at increased risk of thromboembolic stroke when compared to male counterparts despite equal anticoagulation. There were no sex differences in stroke risk among younger cohorts.

It is important to note that despite the statistical significance of increased stroke risk among older female patients (age ≥75 years), the absolute risk reduction between groups was small. These findings may suggest limited clinical significance of female sex as a risk factor in the CHA_2_DS_2_-VASc score given the lack of risk modification in younger cohorts (age <75 years) and the modest risk modification in older patients (age ≥75 years).

With over 2 million patients, this study represents the largest known investigation of female sex as a risk modifier vs risk factor for stroke risk. The large population size included in this study decreases the chance of random error contributing to our results. These results are consistent with previous smaller studies investigating this topic. Nielsen et al stratified the effect of female sex on thromboembolic stroke risk by CHA_2_DS_2_-VASc score, finding female sex to be associated with an increased risk of thromboembolic stroke in all scores except a score of 0 or 3.^10^ Another study by Marzona et al suggested that female sex increases risk of thromboembolic stroke in an age-dependent fashion, with a significant difference in stroke risk existing between female and male patients >65 years old.^14^ In a similar analysis, Mikkelsen et al found that female sex only increased the risk of stroke in non-anticoagulated patients ≥75 years old after controlling for CHA_2_DS_2_-VASc risk factors, with no significant difference found in younger cohorts.^15^ We synthesize the strategies of earlier studies within a single, larger cohort, thereby overcoming limitations of small or heterogeneous populations and enabling cross-group comparison and causal inference within a consistent analytic framework. Our sensitivity analysis supports Nielsen et al in identifying an association between female sex and thromboembolic stroke risk in patients with additional comorbidities present; however, we additionally stratified by age like Marzona et al and Mikkelsen et al, identifying an age-dependency in the association between female sex and thromboembolic stroke risk. Unlike these studies, we found that the age-dependency seems to be present in the absence of multiple comorbidities, with female sex increasing the risk of stroke in these patients only when age ≥75. The age-dependency additionally remains consistent among male and female patients with additional risk factors on anticoagulation, with older female patients having an increased stroke risk despite equal anticoagulation (age ≥75). In this way, our results are essentially a combination of these previous smaller studies finding female sex to be a risk modifier, increasing the risk of stroke only when in the presence of multiple comorbidities or older age (≥75).

These findings support previous research that female sex may serve as a risk modifier, increasing risk of stroke only in patients who have additional risk factors, given the lack of stroke risk difference within the younger age group in the subgroup analysis where all patients had CHA_2_DS_2_-VA score of 0.

Sex differences in cardiac physiology, hormones, and AF diagnosis and treatment patterns may help to explain these findings. Advanced age and female sex have been associated with a higher burden of atrial fibrosis, a known marker of stroke risk in AF patients.^16^ Estrogen is thought to play a role in atrial fibrosis by regulating expression of matrix metalloproteinases, thus helping to maintain extracellular matrix turnover.^17^^,^^18^ Decreased estrogen levels, as occurs after menopause, likely leads to matrix accumulation and atrial fibrosis.^18^^,^^19^ Lastly, female patients are known to be diagnosed with AF later than males, likely due to difference in clinical presentation.^20^ Females are also known to receive fewer ablations and anticoagulation prescriptions compared to males, which contributes to disparities in outcomes.^21^^,^^22^ The results of this study agree with those of previous studies^10^^,^^12^^,^^15^ that the simplified CHA_2_DS_2_-VA score may be sufficient when considering anticoagulation prescription in AF patients. Although female sex appears to be a risk modifier for stroke in patients with multiple comorbidities or age ≥75 years, the CHA_2_DS_2_-VA score still accurately recommends anticoagulation for these patients and may help simplify clinical decision-making.

### Study Limitations

Given the retrospective nature of this study, the findings are associative and do not establish causality. Use of the TriNetX database introduces inherent limitations, including potential coding inaccuracies, misclassification, incomplete data capture, and residual confounding from unmeasured variables. Several variables known to influence stroke risk, such as left atrial size and level of atrial fibrosis, were not available. In our risk factor analysis, there were several limitations related to anticoagulation. We were unable to assess medication adherence or INR values at the time of stroke for those on warfarin. Anticoagulation rates in the study population were low across all cohorts, including in the higher-risk age groups (≥75). Additionally, there are known sex differences in anticoagulation prescribing patterns, with females often receiving less anticoagulation, which we attempted to control for in propensity matching. In addition, formal testing of the proportional hazards assumption (eg, Schoenfeld residuals) was not available within the TriNetX platform, and although visual inspection of Kaplan-Meier curves did not suggest major violations, this remains a limitation.

## Conclusions

For patients without multiple comorbidities and with moderate age (<75), female sex was not associated with an increased thromboembolic stroke risk. Therefore, to improve the overall quality of clinical treatment and anticoagulation determinations, the heterogeneity of female sex should be taken into consideration by physicians.

Perspectives **COMPETENCY IN MEDICAL KNOWLEDGE:** Female AF patients <75 without multiple risk factors are a low-risk group for thromboembolic stroke, thus anticoagulation may not be necessary.

**TRANSLATIONAL OUTLOOK:** Conduct a prospective study analyzing difference in atrial fibrosis formation in males vs females stratified by CHA_2_DS_2_-VA score. Explore sex-specific cellular pathways that may contribute to greater severity and increased stroke risk in female patients compared with male patients.

## Funding support and author disclosures

Dr Marrouche has received consulting fees from 10.13039/100007497Biosense Webster, 10.13039/100008497Boston Scientific, and 10.13039/100007330AtriCure; has served as a speaker for 10.13039/100000046Abbott, 10.13039/100007497Biosense Webster, 10.13039/100007330AtriCure, and 10.13039/100004339Sanofi; has received research support from 10.13039/100000046Abbott, 10.13039/100004374Medtronic, 10.13039/100007497Biosense Webster, Siemens GE, 10.13039/100008497Boston Scientific, 10.13039/100004339Sanofi, and Samsung; has a family member as the CEO of Cardiac Designs; and is the founder of Marrek, being named in a patent issued for MRI fibrosis imaging and being a previous shareholder of Cardiac Designs. Dr Pandey has served on an advisory board for Novartis (completed). All other authors have reported that they have no relationships relevant to the contents of this paper to disclose.
