# Supplementary material for: Female Sex Is Not a Uniform Risk Factor in Atrial Fibrillation
Source: JACC Adv. 2026 Jun 3;5(6):102826. doi: 10.1016/j.jacadv.2026.102826 (PMC13309320; doi:10.1016/j.jacadv.2026.102826)
Supplement: Supplemental Material [file mmc1.pdf]

Supplemental Information:

Supplemental Table 1: Baseline Characteristics < 65 Cohort

**CHA2DS2-VA: 0**	**Before Propensity Matching**			**After Propensity Matching**		
**Variable (ICD-10), percent (n)**	**Female**	**Male**	**p-value**	**Female**	**Male**	**p-value**
**Percent of cohort (n)**	38.7 (37,993)	61.3 (60,142)		50.0 (37,991)	50.0 (37,991)	
**Current age (SD)**	48.0 (12.8)	48.5 (12.3)	<0.001	48.0 (12.8)	48.0 (12.8)	0.901
**Atherosclerotic heart disease (I25.1)**	0.9 (360)	1.1 (691)	0.003	0.9 (358)	1.0 (385)	0.320
**Ischemic cardiomyopathy (I25.5)**	0.0 (10)	0.0 (28)	0.117	0.0 (10)	0.0 (10)	1
**Tobacco use (Z72.0)**	1.8 (671)	1.7 (999)	0.215	1.8 (671)	1.7 (640)	0.388
**OSA (G47.33)**	2.8 (1,066)	3.0 (1,793)	0.111	2.8 (1,066)	2.8 (1,052)	0.758
**BMI (SD)**	28.8 (8.0)	28.3 (6.4)	<0.001	28.8 (8.0)	28.2 (6.4)	<0.001
**CHA2DS2-VA: 1 or 2**	**Before Propensity Matching**			**After Propensity Matching**		
**Variable (ICD-10), percent (n)**	**Female**	**Male**	**p-value**	**Female**	**Male**	**p-value**
**Percent of cohort (n)**	35.0 (29,801)	65.0 (53,606)		50.0 (28,889)	50.0 (228,889)	
**Current age (SD)**	53.4 (10.0)	53.7 (9.5)	<0.001	53.4 (10.0	53.4 (9.9)	0.885
**Atherosclerotic heart disease (I25.1)**	2.6 (749)	4.1 (2,182)	<0.001	2.6 (7499)	2.6 (739)	0.793

**Ischemic cardiomyopathy (I25.5)**	0.1 (18)	0.2 (92)	<0.001	0.1 (18)	0.0 (13)	0.369
**Tobacco use (Z72.0)**	1.9 (551)	2.0 (1,066)	0.349	1.9 (550)	2.0 (567)	0.608
**OSA (G47.33)**	4.9 (1,427)	5.2 (2,786)	0.068	4.9 (1,427)	4.9 (1,427)	1
**BMI (SD)**	32.4 (9.0)	31.0 (7.3)	<0.001	32.5 (9.0)	30.9 (7.3)	<0.001
**Anticoagulated: CHA2DS2-VA: 1-6**	**Before Propensity Matching**			**After Propensity Matching**		
**Variable (ICD-10), percent (n)**	**Female**	**Male**	**p-value**	**Female**	**Male**	**p-value**
**Percent of cohort (n)**	39.1 (3,714)	60.9 (5,780)		50.0 (3,269)	50.0 (3,269)	
**Current age (SD)**	56.8 (7.2)	57.4 (6.8)	<0.001	56.9 (7.1)	57.0 (7.0)	0.573
**Hypertension (I10)**	69.5 (2,578)	67.8 (3,916)	0.083	69.1 (2,507)	68.8 (2,495)	0.761
**Type 2 Diabetes Mellitus (E11)**	39.0 (1,445)	34.6 (1,995)	<0.001	38.1 (1,284)	37.5 (1,361)	0.578
**Atherosclerotic heart disease (I25.1)**	27.1 (1,004)	32.4 (1,872)	<0.001	27.3 (989)	25.5 (926)	0.093
**Ischemic cardiomyopathy (I25.5)**	3.0 (111)	6.3(365)	<0.001	3.1 (111)	2.9 (104)	0.628
**Systolic heart failure (I150.2)**	11.3 (418)	16.8 (969)	<0.001	11.5 (417)	10.6 (386)	0.246
**Diastolic heart failure (I50.3)**	12.7 (470)	9.5 (546)	<0.001	12.0 (435)	11.2 (407)	0.305

**Prior Myocardial Infarction (I21)**	11.9 (442)	14.7 (847)	<0.001	11.9 (432)	10.7 (389)	0.111
**Prior TIA (G45)**	61.2 (2,269)	57.8 (3,336)	0.001	61.0 (2,212)	58.0 (2,106)	0.011
**Other peripheral vascular disease (I73)**	11.4 (424)	10.1 (585)	0.045	11.2 (407)	9.8 (355)	0.046
**Tobacco use (Z72.0)**	12.8 (474)	12.0 (692)	0.249	12.4 (451)	11.2(408)	0.118
**OSA (G47.33)**	27.3 (1,014)	28.2 (1,626)	0.385	27.3 (989)	26.5 (960)	0.442
**BMI (SD)**	34.5 (9.6)	32.7 (7.9)	<0.001	34.4 (9.5)	32.8 (8.0)	<0.001
**Warfarin**	16.1(598)	12.3 (710)	<0.001	15.3 (554)	14.7 (534)	0.511
**Apixaban**	18.8 (697)	15.2 (880)	<0.001	18.2 (662)	18.4 (669)	0.832
**Rivaroxaban**	8.0 (295)	6.7 (389)	0.025	7.7 (280)	7.1 (256)	0.281
**Dabigatran**	0.3 (10)	0.2 (10)	0.318	0.3 (10)	0.3 (10)	1
**Edoxaban**	0.3 (10)	0.2 (10)	0.318	0.3 (10)	0.3 (10)	1

Table 1: Baseline characteristics in AF patients < 65 years before and after propensity matching.

Supplemental Table 2: Baseline Characteristics 65-74 Cohort

CHA2DS2-VA: 1	Before Propensity Matching			After Propensity Matching		
**Variable (ICD-10), percent (n)**	**Female**	**Male**	**p-value**	**Female**	**Male**	**p-value**
**Percent of cohort (n)**	39.4 (24,702)	60.6 (37,630)		50.0 (24,699)	50.0 (24,699)	
**Current age (SD)**	69.7 (2.9)	69.6 (2.8)	0.160	69.7 (2.9)	69.7 (2.9)	0.815
**Atherosclerotic heart disease (I25.1)**	2.0 (482)	3.3 (1,244)	<0.001	1.9 (481)	2.0 (482)	0.974
**Ischemic cardiomyopathy (I25.5)**	0.0 (10)	0.1 (51)	<0.001	0.0 (10)	0.0 (10)	1
**Tobacco use (Z72.0)**	1.1 (283)	1.3 (476)	0.184	1.1 (280)	1.1 (280)	1
**OSA (G47.33)**	2.5 (625)	2.4 (917)	0.463	2.5 (622)	2.5 (608)	0.686
**BMI (SD)**	27.8 (7.3)	27.9 (5.6)	0.750	27.8 (7.3)	27.9 (5.6)	0.588
**CHA2DS2-VA: 2 or 3**	**Before Propensity Matching**			**After Propensity Matching**		
**Variable (ICD-10), percent (n)**	**Female**	**Male**	**p-value**	**Female**	**Male**	**p-value**
**Percent of cohort (n)**	37.0 (34,817)	63.0 (59,243)		50.0 (34,623)	50.0 (34,623)	
**Current age (SD)**	69.9 (2.8)	69.8 (2.8)	<0.001	69.9 (2.8)	69.9 (2.8)	0.941
**Atherosclerotic heart disease (I25.1)**	4.4 (1,530)	7.9 (4,679)	<0.001	4.4 (1,530)	4.4 (1,526)	0.941

**Ischemic cardiomyopathy (I25.5)**	0.1 (29)	0.3 (204)	<0.001	0.1 (29)	0.1 (29)	1
**Tobacco use (Z72.0)**	1.4 (492)	1.4 (810)	0.574	1.4 (491)	1.4 (478)	0.674
**OSA (G47.33)**	3.7 (1,269)	4.1 (2,398)	0.002	3.7 (1,268)	3.6 (1,260)	0.871
**BMI (SD)**	30.3 (8.0)	29.3 (6.1)	<0.001	30.3 (8.0)	29.3 (6.2)	<0.001
**Anticoagulated: CHA2DS2-VA: 1-7**	**Before Propensity Matching**			**After Propensity Matching**		
**Variable (ICD-10), percent (n)**	**Female**	**Male**	**p-value**	**Female**	**Male**	**p-value**
**Percent of cohort (n)**	38.7 (4,486)	61.3 (7,093)		50.0 (4,413)	50.0 (4,413)	
**Current age (SD)**	70.2 (2.8)	70.1 (2.8)	0.022	70.2 (2.8)	70.2 (2.8)	0.459
**Hypertension (I10)**	74.7 (3,344)	72.8 (5,144)	0.023	74.3 (3,281)	74.1 (3,270)	0.789
**Type 2 Diabetes Mellitus (E11)**	39.4 (1,766)	36.9 (2,607)	0.006	38.8 (1,714)	37.9 (1,673)	0.369
**Atherosclerotic heart disease (I25.1)**	31.1 (1,394)	39.8 (2,812)	<0.001	31.2 (1,376)	29.8 (1,316)	0.165
**Ischemic cardiomyopathy (I25.5)**	2.8 (126)	6.4 (456)	<0.001	2.9 (126)	2.6 (113)	0.394
**Systolic heart failure (I150.2)**	7.1 (320)	12.2 (864)	<0.001	7.3 (320)	6.3 (277)	0.068
**Diastolic heart failure (I50.3)**	12.1 (544)	9.0 (638)	<0.001	11.5 (508)	10.2 (450)	0.047

**Prior Myocardial Infarction (I21)**	11.0 (494)	13.1 (929)	0.001	10.8 (478)	10.0 (442)	0.210
**Prior TIA (G45)**	60.4 (2,705)	57.1 (4,038)	<0.001	60.2 (2,657)	56.8 (2,506)	0.001
**Other peripheral vascular disease (I73)**	13.4 (599)	12.7 (898)	0.293	12.9 (571)	11.9 (524)	0.129
**Tobacco use (Z72.0)**	10.0 (450)	10.4 (735)	0.549	9.9 (439)	9.3 (410)	0.295
**OSA (G47.33)**	20.5 (918)	22.0 (1,555)	0.057	20.4 (899)	19.8 (874)	0.507
**BMI (SD)**	32.4 (8.7)	30.6 (6.6)	<0.001	32.3 (8.7)	30.8 (6.6)	<0.001
**Warfarin**	10.3(462)	9.8 (695)	0.396	10.2 (452)	9.3 (411)	0.142
**Apixaban**	16.4 (735)	14.8 (1,044)	0.014	16.3 (718)	16.1 (711)	0.840
**Rivaroxaban**	7.3 (329)	6.1 (432)	0.009	7.2 (316)	6.7 (297)	0.426
**Dabigatran**	0.2 (10)	0.2 (14)	0.771	0.2 (10)	0.2 (10)	1
**Edoxaban**	0.3 (13)	0.3 (22)	0.842	0.3 (13)	0.3 (12)	1

Table 2: Baseline characteristics in AF patients aged 65-74 years before and after propensity matching.

Supplemental Table 3: Baseline Characteristics in ≥ 75 Cohort

CHA2DS2-VA: 2	Before Propensity Matching			After Propensity Matching		
**Variable (ICD010), percent (n)**	**Female**	**Male**	**p-value**	**Female**	**Male**	**p-value**
**Percent of cohort (n)**	47.7 (68,371)	53.3 (74,935)		50.0 (63,574)	50.0 (63,574)	
**Current age (SD)**	85.3 (5.2)	84.3 (5.4)	<0.001	85.0 (5.2)	85.0 (5.2)	0.880
**Atherosclerotic heart disease (I25.1)**	1.9 (1,298)	3.9 (2,938)	<0.001	2.0 (1,298)	2.0 (1,296)	0.968
**Ischemic cardiomyopathy (I25.5)**	0.1 (36)	0.2 (138)	<0.001	0.1 (36)	0.1 (35)	0.906
**Tobacco use (Z72.0)**	0.3 (199)	0.3 (224)	0.784	0.3 (195)	0.3 (173)	0.251
**OSA (G47.33)**	0.8 (531)	1.3 (979)	<0.001	0.8 (531)	0.8 (527)	0.902
**BMI (SD)**	26.0 (6.2)	26.5 (5.0)	<0.001	26.1 (6.3)	26.4 (5.0)	<0.001
**CHA2DS2-VA: 3 or 4**	**Before Propensity Matching**			**After Propensity Matching**		
**Variable (ICD010), percent (n)**	**Female**	**Male**	**p-value**	**Female**	**Male**	**p-value**
**Percent of cohort (n)**	49.5 (281,078)	50.5 (287,031)		50.0 (240,294)	50.0 (240,294)	
**Current age (SD)**	85.2 (5.1)	84.0 (5.3)	<0.001	84.6 (5.1)	84.6 (5.1)	0.625
**Atherosclerotic heart disease (I25.1)**	4.5 (12,289)	9.2 (25,775)	<0.001	5.1 (12,289)	5.1 (12,302)	0.932

**Ischemic cardiomyopathy (I25.5)**	0.1 (325)	0.5 (1,444)	<0.001	0.1 (325)	0.1 (330)	0.845
**Tobacco use (Z72.0)**	0.3 (787)	0.4 (1,106)	<0.001	0.3 (770)	0.3 (760)	0.798
**OSA (G47.33)**	1.4 (3,787)	2.5 (6,901)	<0.001	1.6 (3,787)	1.6 (3,861)	0.394
**BMI (SD)**	27.4 (6.6)	27.6 (5.2)	<0.001	27.6 (6.7)	27.5 (5.2)	0.006
**Warfarin**	3.7 (10,127)	4.4 (12,252)	<0.001	4.2 (10,003)	4.0 (9,268)	0.006
**Apixaban**	7.7 (21,075)	7.5 (20,961)	0.017	7.7 (18,440)	7.6 (18,283)	0.394
**Rivaroxaban**	2.6 (7,032)	2.9 (8,157)	<0.001	2.8 (6,658)	2.7 (6,531)	0.262
**Dabigatran**	0.0 (119)	0.1 (150)	0.080	0.0 (115)	0.0 (96)	0.191
**Edoxaban**	0.1 (228)	0.1 (222)	0.643	0.1 (199)	0.1 (183)	0.413
**Anticoagulated: CHA2DS2-VA: 2-7**	**Before Propensity Matching**			**After Propensity Matching**		
**Variable (ICD-10), percent (n)**	**Female**	**Male**	**p-value**	**Female**	**Male**	**p-value**
**Percent of cohort (n)**	48.7 (40,069)	51.3 (42,229)		50.0 (33,209)	50.0 (33,209)	
**Current age (SD)**	85.5 (4.9)	84.4 (5.1)	<0.001	85.0 (5.0)	84.9 (5.0)	0.549
**Hypertension (I10)**	76.5 (30,399)	74.1 (31,035)	<0.001	73.5 (24,420)	73.8 (24,519)	0.383
**Type 2 Diabetes Mellitus (E11)**	31.1 (12,347)	33.9 (14,189)	<0.001	31.7 (10,516)	32.1 (10,649)	0.268
**Atherosclerotic heart disease (I25.1)**	29.9 (11,892)	43.9 (18,385)	<0.001	34.6 (11,490)	34.3 (11,420)	0.568

**Ischemic cardiomyopathy (I25.5)**	2.1 (832)	6.1 (2,555)	<0.001	2.5 (831)	2.5 (837)	0.882
**Systolic heart failure (I150.2)**	6.4 (2,538)	9.8 (4,123)	<0.001	7.1 (2,346)	6.8 (2,271)	0.253
**Diastolic heart failure (I50.3)**	13.3 (5,308)	10.3 (4,300)	<0.001	11.3 (3,743)	10.9 (3,614)	0.111
**Prior Myocardial Infarction (I21)**	9.3 (3,713)	11.4 (4,778)	<0.001	9.8 (3,244)	9.5 (3,142)	0.179
**Prior TIA (G45)**	58.8 (23,290)	57.0 (23,866)	<0.001	58.2 (19,312)	55.9 (218,559)	<0.001
**Other peripheral vascular disease (I73)**	12.5 (4,987)	14.7 (6,158)	<0.001	13.1(4,351)	12.8 (4,264)	0.315
**Tobacco use (Z72.0)**	2.4 (955)	2.9 (1,196)	0<0.001	2.6 (861)	2.4 (802)	0.143
**OSA (G47.33)**	10.0 (3,988)	15.2 (6,370)	<0.001	11.6 (3,842)	11.5 (3,807)	0.671
**BMI (SD)**	28.6 (6.9)	28.5 (5.4)	0.001	28.9 (7.0)	28.3 (5.3)	<0.001
**Warfarin**	10.6 (4,207)	11.3 (4,735)	0.396	11.1 (3,692)	10.8 (3,599)	0.248
**Apixaban**	11.5 (4,568)	11.3 (4,734)	0.411	11.6 (3,837)	11.4 (3,775)	0.450
**Rivaroxaban**	4.7 (1,873)	4.6 (1,910)	0.311	4.7 (1,574)	4.4 (1,469)	0.051
**Dabigatran**	0.1 (35)	0.1 (38)	0.896	0.1 (32)	0.1 (32)	0.900
**Edoxaban**	0.2 (84)	0.1 (61)	0.026	0.2 (65)	0.2 (55)	0.361

Table 3: Baseline characteristics in AF patients aged ≥ 75 years before and after propensity matching.

Supplemental Table 4: ICD-10 Demographics

**Condition**	**ICD-10 Codes**
Atrial Fibrillation	I48.0, I48.1, I48.2, I48.91
Diabetes Mellitus	E11.x
Hypertension	I10.x
Coronary Artery Disease	I25.10
Systolic Heart Failure	I50.2
Diastolic Heart Failure	I50.3
Ischemic Cardiomyopathy	I25.5
History of Myocardial Infarction	I21.x
History of Cerebral Infarction	I63.x
History of TIA	G45.x
Other Peripheral Vascular Diseases	I73.x
Obstructive Sleep Apnea	G47.33
Tobacco Use	Z72.0

Table 4: ICD-10 codes used to define key cardiovascular and systemic comorbidities in the study population for matching and adjustment, including diabetes, hypertension, hyperlipidemia, coronary artery disease, and other relevant chronic conditions.

Supplemental Table 5: Outcome ICDs

**Outcome**	**ICD-10-CM Codes**
Cerebral Infarction	I63.x
Arterial Embolism and Thrombosis	I74.x

Table 5: ICD-10 codes used to identify primary cardiovascular outcomes of interest, including cerebral infarction and arterial embolism.

Supplemental Table 6: Other Exclusion ICDs

**Condition**	**ICD-10 Code(s)**
Nonrheumatic Mitral Valve Insufficiency	I34.0
Rheumatic Mitral Valve Diseases	I05.x
Rheumatic Mitral Stenosis	I05.0
Other Rheumatic Mitral Valve Diseases	I05.8
Nonrheumatic Mitral Valve Stenosis	I34.2

Table 6: ICD-10 codes used to exclude patients with atrial fibrillation secondary to valvular disorders, ensuring a cohort of entirely nonvalvular atrial fibrillation patients.

Supplemental Table 7: Anticoagulation RXNORM

**Medication**	**RXNORM**
Warfarin	11289
Apixaban	1364430
Rivaroxaban	1114195
Dabigatran	1546356
Edoxaban	1599538

Table 7: RXNORM codes used to identify anticoagulation prescriptions within cohorts.

Supplemental Figure 1:

1a.

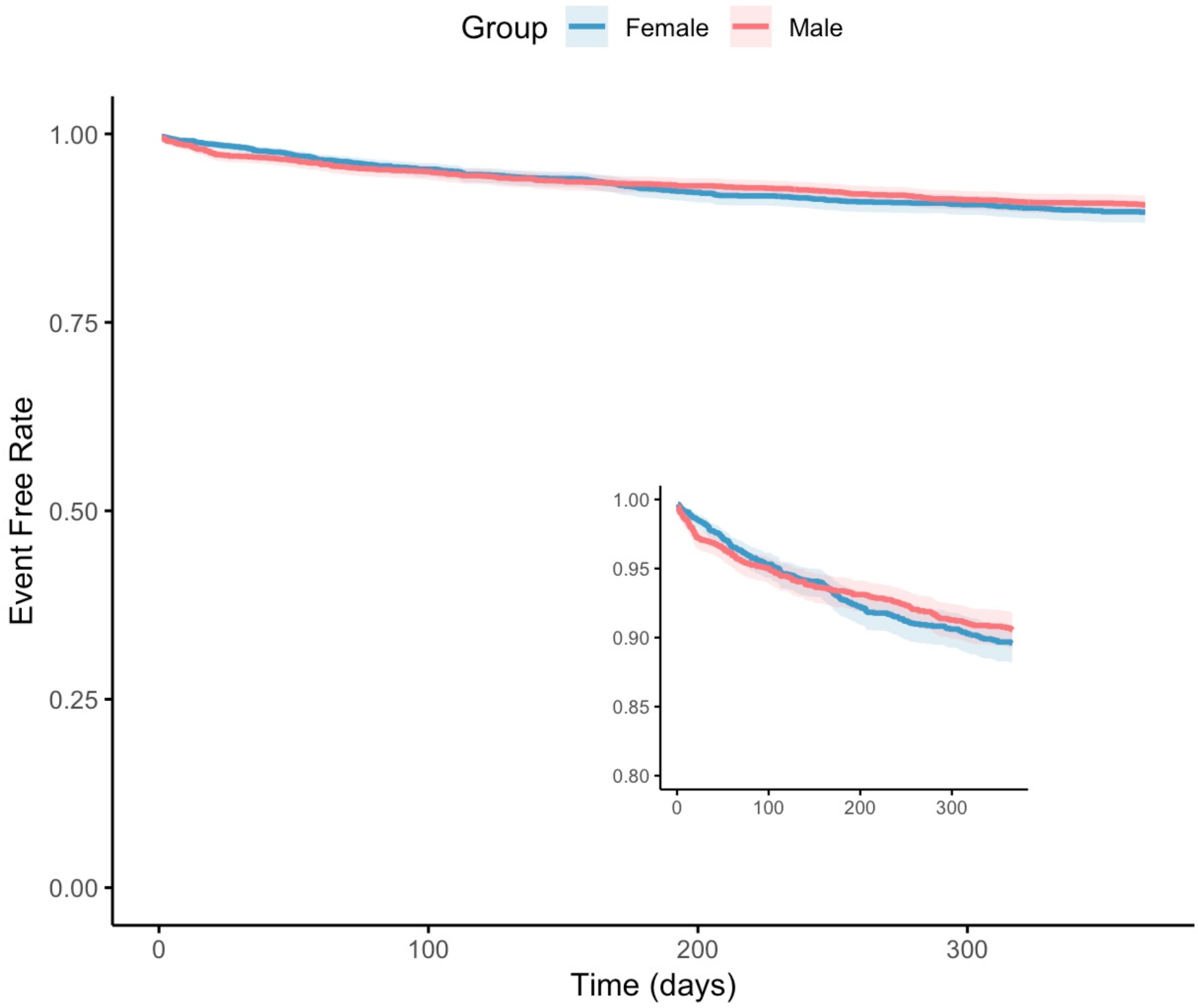

Kaplan-Meier survival analysis in <65 years cohort in sensitivity analysis including only patients on current anticoagulation. There was no significant difference in 1-year stroke risk between male and female cohorts (HR: 1.090, 95% CI: 0.895-1.327, p=0.393).

1b.

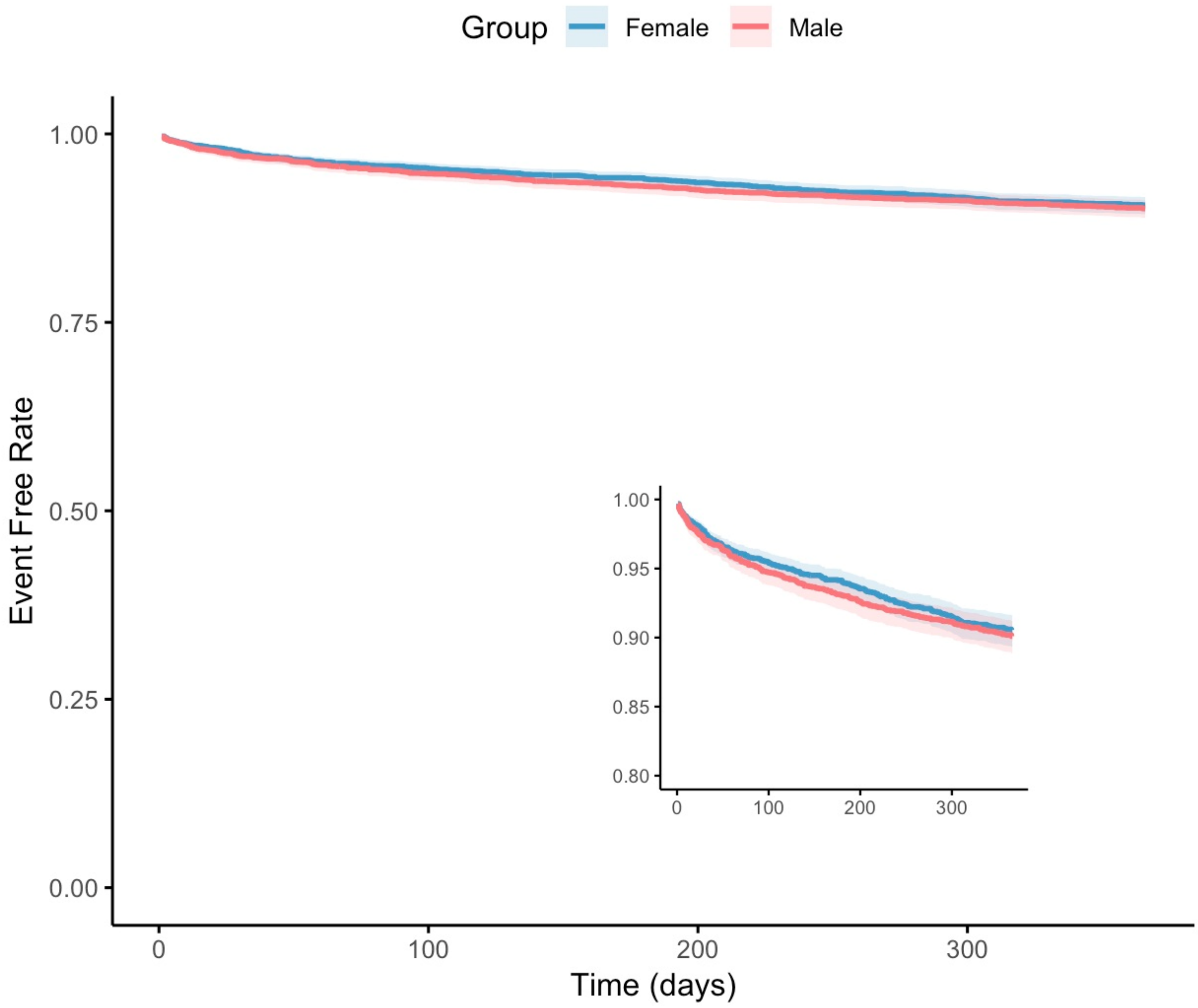

Kaplan-Meier survival analysis in 65-74 years cohort in sensitivity analysis including only anticoagulated patients. There was no significant difference in 1-year stroke risk between male and female patients (HR: 0.944, 95% CI: 0.792-1.125, p=0.517).

1c.

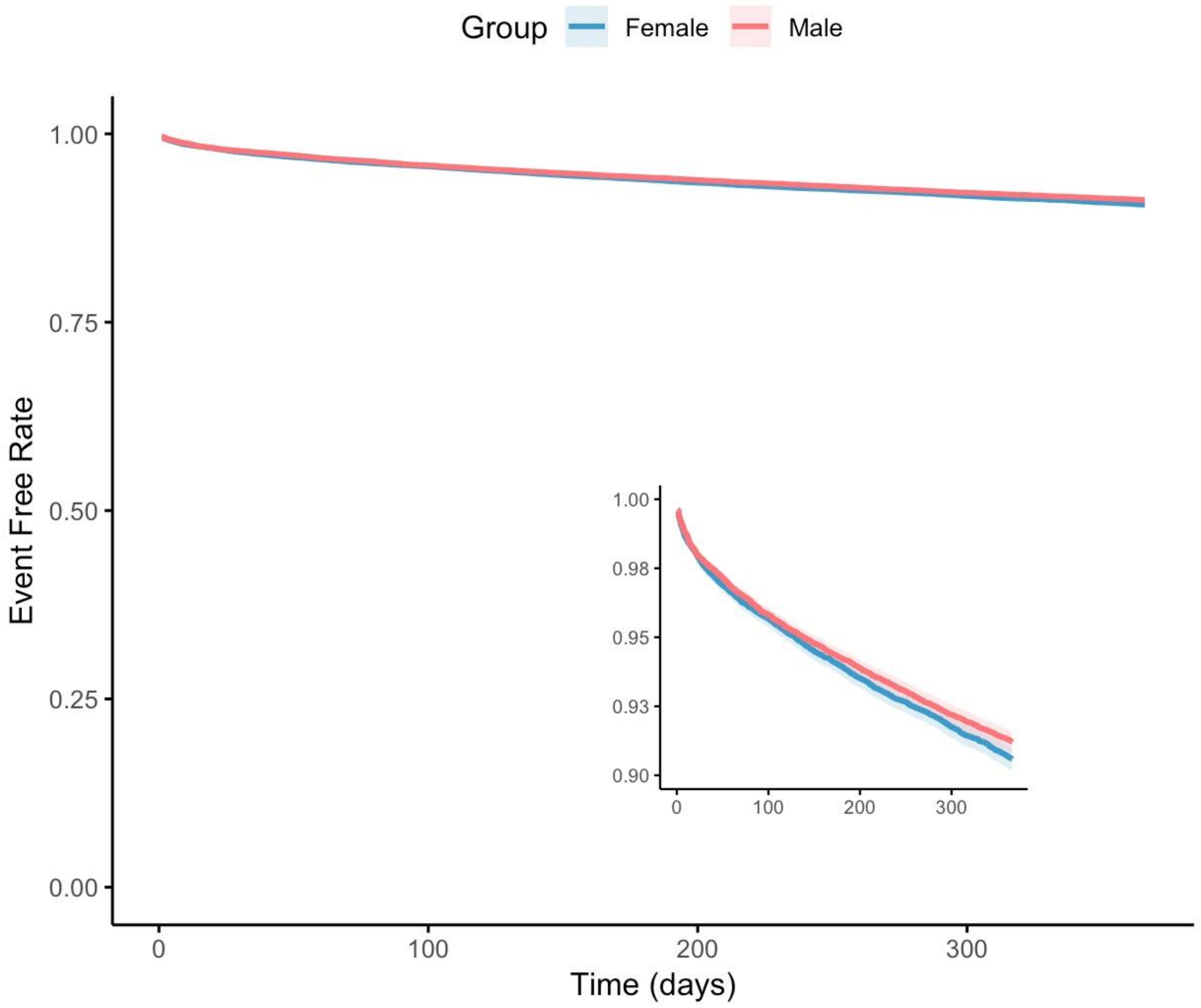

Kaplan-Meier survival analysis in ≥75 years cohort in sensitivity analysis including only patients on current anticoagulation. Despite equal anticoagulation between groups, female patients in this age group were at significantly increased risk of stroke during a 1-year period (HR: 1.071, 95% CI: 1.004-1.143, p=0.037).
